# CT imaging features of pulmonary SMARCA4-deficient undifferentiated carcinoma: a retrospective case series

**DOI:** 10.3389/fonc.2026.1822031

**Published:** 2026-05-18

**Authors:** Yujian Liu, Tianjiao Lv, Limei Han, Yuan Li

**Affiliations:** 1 Department of Radiology, Zigong First People’s Hospital, Zigong, Sichuan, China; 2 Department of Ultrasound, Zigong Fourth People’s Hospital, Zigong, Sichuan, China

**Keywords:** aggressive imaging features, computed tomography, lung, SMARCA4 deficiency, undifferentiated carcinoma

## Abstract

**Background:**

Pulmonary SMARCA4-deficient undifferentiated carcinoma is a rare and highly aggressive thoracic malignancy characterized by rapid progression, poor prognosis, and nonspecific imaging features. Although its pathological and molecular characteristics are increasingly recognized, its CT imaging features remain insufficiently characterized. This study aimed to systematically describe the CT imaging features of this entity and to summarize coexisting imaging findings that may assist radiologic suspicion.

**Methods:**

This retrospective study included 15 patients with pathologically and immunohistochemically confirmed pulmonary SMARCA4-deficient undifferentiated carcinoma. All patients underwent baseline chest CT before any antitumor treatment, including non-contrast and contrast-enhanced scans. Two experienced thoracic radiologists, blinded to pathological results, independently evaluated tumor location, size, morphology, margins, intratumoral necrosis or cavitation, enhancement characteristics, invasion of adjacent structures, lymphadenopathy, pleural involvement, and distant metastases. Interobserver agreement was assessed using Cohen’s κ, and analyses were descriptive.

**Results:**

Patients were predominantly middle-aged to elderly men, most with a smoking history, and the majority presented with locally advanced or advanced-stage disease. On CT, tumors typically appeared as solid masses with a median maximum diameter of 3.7 cm (range, 0.6–9.5 cm), nearly half of which were centrally located. Aggressive imaging features were common, including spiculated margins (93.3%), intratumoral necrosis (86.7%), and cavitation (46.7%). Contrast-enhanced CT frequently demonstrated heterogeneous moderate-to-marked enhancement. Invasion of adjacent structures was prevalent, including bronchial involvement (86.7%), vascular invasion (80.0%), and mediastinal invasion (66.7%). Lymph node metastases were observed in 86.7% of cases, often as conglomerated lymphadenopathy (73.3%). Key imaging features showed good-to-excellent interobserver agreement. All cases demonstrated loss of SMARCA4 (BRG1) nuclear expression with retained INI-1.

**Conclusion:**

Pulmonary SMARCA4-deficient undifferentiated carcinoma showed a reproducible aggressive CT imaging profile in this cohort, characterized by marked heterogeneity, necrosis or cavitation, multi-structure invasion, and conglomerated lymphadenopathy. Although these findings are not individually specific, their co-occurrence may help raise suspicion for this entity over other aggressive thoracic malignancies with overlapping imaging features and support earlier targeted immunohistochemical confirmation. Further multicenter studies are needed to validate this CT imaging profile and clarify its clinical relevance.

## 1 Introduction

Pulmonary SMARCA4-deficient undifferentiated carcinoma is a rare and highly aggressive thoracic malignancy that has been increasingly recognized in recent years (1–4). It predominantly affects middle-aged and older individuals, most of whom have a history of smoking. The disease is characterized by rapid clinical progression, poor prognosis, and a high prevalence of advanced-stage presentation at diagnosis. Pathologically, it is defined by loss of nuclear SMARCA4 (BRG1), a key subunit of the SWI/SNF chromatin remodeling complex, often accompanied by marked dedifferentiation. Immunohistochemically, these tumors frequently lack or show only focal expression of epithelial markers, resulting in substantial overlap with sarcomatoid carcinoma, poorly differentiated non–small cell lung cancer, neuroendocrine tumors, and other aggressive thoracic malignancies. Although advances in molecular pathology have improved disease characterization, accurate diagnosis still relies on multidisciplinary integration of clinical, radiologic, histopathologic, and molecular information (1, 2, 5, 6).

Previous studies, largely consisting of case reports, small case series, and clinicopathologic analyses, have described aggressive imaging features such as necrosis, heterogeneous enhancement, and invasion of adjacent structures (2, 6–10). However, these findings have typically been reported in a fragmented manner, and the relative importance, co-occurrence, and potential diagnostic implications of combined imaging features remain insufficiently characterized. As a result, pulmonary SMARCA4-deficient undifferentiated carcinoma may still be misinterpreted as other high-grade pulmonary malignancies in routine clinical practice, potentially delaying appropriate immunohistochemical confirmation and clinical management (2, 3, 8, 9).

CT plays a central role in the detection, staging, and management of thoracic malignancies. Beyond individual imaging findings, a systematic characterization of coexisting CT features and their reproducibility may provide more practical value for radiologic recognition. Therefore, this study aimed to systematically analyze baseline CT imaging findings in 15 pathologically confirmed cases of pulmonary SMARCA4-deficient undifferentiated carcinoma, to characterize a recognizable imaging profile defined by the co-occurrence of multiple aggressive CT features, and to evaluate their interobserver reproducibility, with the goal of providing practical imaging clues to facilitate radiologic suspicion and support multidisciplinary diagnostic decision-making.

## 2 Methods

### 2.1 Study design, clinical data collection, and follow-up

This was a single-center retrospective study. By searching cases archived in the institutional picture archiving and communication system (PACS) between January 2021 and December 2025, a total of 15 consecutive patients with pulmonary SMARCA4-deficient undifferentiated carcinoma were identified. All diagnoses were confirmed by histopathological examination and immunohistochemical analysis. All patients underwent chest CT at initial presentation, and none had received any antitumor treatment prior to imaging. The study protocol was approved by the institutional ethics committee, and the requirement for informed consent was waived owing to the retrospective nature of the study.

Clinical data collected included patient age, sex, smoking history, Eastern Cooperative Oncology Group (ECOG) performance status at initial diagnosis, clinical tumor–node–metastasis (TNM) stage, and the presence of distant metastases at baseline. Tumor staging was determined according to the 8th edition of the American Joint Committee on Cancer (AJCC) lung cancer staging system, based on imaging findings at initial presentation and recorded as clinical TNM (cTNM) stage.

The assessment of distant metastases was based on chest CT in combination with available concurrent or subsequent imaging studies, including abdominal CT, bone imaging, or PET-CT when clinically indicated. Due to the retrospective nature of the study, imaging protocols were not fully standardized across all patients.

For descriptive follow-up assessment, synchronous metastasis was defined as distant metastasis detected on baseline staging imaging at initial diagnosis, whereas metachronous metastasis referred to newly identified distant lesions during follow-up after the initial imaging evaluation.

All cases were diagnosed using surgical resection specimens or biopsy samples. Pathologic diagnosis was established based on routine histomorphologic evaluation and immunohistochemical analysis. Immunohistochemical staining included, but was not limited to, SMARCA4 (BRG1), INI-1, pancytokeratin (P-CK), TTF-1, Napsin A, p40, CK5/6, neuroendocrine markers, and NUT, and was performed to confirm SMARCA4 deficiency and to exclude other undifferentiated tumor entities.

All patients were followed up through outpatient visits, inpatient medical records, and telephone interviews. Follow-up was continued until December 2025 or death, whichever occurred first. Treatment modalities and survival status were recorded for descriptive outcome analysis.

### 2.2 CT acquisition protocol

All patients underwent baseline chest CT examinations using a SOMATOM Force CT scanner (Siemens Healthineers, Germany), including both non-contrast and contrast-enhanced acquisitions. The scanning range extended from the thoracic inlet to the diaphragm.

Non-contrast CT scanning parameters were as follows: tube voltage of 100–120 kVp, automatic tube current modulation, slice thickness of 1.0–1.5 mm, slice interval of 1.0–1.5 mm, and a reconstruction matrix of 512 × 512. Images were reconstructed and evaluated using standard lung and mediastinal window settings. The lung window width was approximately 1200–1600 HU with a window level of −500 to −700 HU, and the mediastinal window width was approximately 350–450 HU with a window level of 40–60 HU.

For contrast-enhanced CT, a nonionic iodinated contrast agent was administered intravenously via an antecubital vein at an injection rate of 2.5–3.0 mL/s, with the dose adjusted according to body weight (approximately 1.2–1.5 mL/kg), followed by a saline flush. Image acquisition was performed at a delay of approximately 25–70 seconds, corresponding to the arterial or parenchymal phase. Contrast-enhanced images were reconstructed with thin slices and used to evaluate tumor enhancement patterns and relationships with adjacent structures.

### 2.3 CT image analysis and imaging definitions

All CT images were transferred to the institutional picture archiving and communication system (PACS) for post-processing and analysis.

All CT images were reviewed on a picture archiving and communication system (PACS) workstation. Two experienced thoracic radiologists, who were blinded to pathological results and clinical outcomes, independently evaluated all imaging studies. Any discrepancies were resolved by consensus. Interobserver agreement for major imaging features was assessed using Cohen’s κ statistics.

Tumor location was determined according to the relationship between the lesion center and the hilar structures. Tumors were classified as central when the lesion center was located in the proximal segmental bronchi or adjacent to the pulmonary hilum, and as peripheral otherwise. Tumor size was defined as the maximum diameter measured on axial images or multiplanar reconstructions.

Morphologic features included tumor margins and internal characteristics. Spiculation was defined as radiating linear strands extending from the tumor margin into the surrounding lung parenchyma. Tumor necrosis was defined as a low-attenuation area within the tumor that showed no appreciable enhancement on contrast-enhanced CT. Cavitation was defined as a gas-containing or air–fluid level space surrounded by a definable wall within the tumor.

Tumor enhancement was qualitatively assessed on contrast-enhanced CT according to the degree of enhancement of the solid tumor component and categorized as mild, moderate, or marked, with reference to attenuation differences relative to surrounding structures. The homogeneity of enhancement was also recorded. Given the retrospective design of the study and variations in scanning parameters and enhancement timing among patients, quantitative attenuation measurements were not standardized, and enhancement assessment was primarily based on visual evaluation.

Mediastinal invasion was defined as extensive contact between the tumor and mediastinal structures with obliteration of the intervening fat plane, loss of the interface, or deformation/compression of mediastinal organs. Vascular invasion was defined as tumor encasement of an adjacent major vessel involving more than 180° of the vessel circumference, or associated luminal narrowing or contour irregularity. Bronchial invasion was defined as bronchial narrowing, truncation, or encasement by tumor tissue.

Lymph node assessment was based on short-axis diameter criteria. Hilar or mediastinal lymph nodes with a short-axis diameter of ≥10 mm were considered enlarged. Conglomerated lymph nodes were defined as multiple enlarged lymph nodes with indistinct margins forming a contiguous mass. Pleural involvement included direct tumor extension to the pleura, pleural thickening, or adjacent reactive pleural changes.

The presence of distant metastases was determined based on chest CT in conjunction with concurrent or subsequent imaging studies, including involvement of the bone, adrenal glands, liver, or other organs.

### 2.4 Statistical analysis

This study was a single-center retrospective descriptive analysis. Continuous variables were summarized as median (range), and categorical variables were expressed as counts and percentages [n (%)]. All imaging analyses were based on baseline CT examinations obtained at initial diagnosis prior to any antitumor treatment.

To evaluate interobserver agreement, the two thoracic radiologists independently assessed key imaging features, including tumor location, intratumoral necrosis, mediastinal invasion, vascular invasion, and conglomerated lymph nodes. Cohen’s κ statistics were used to assess agreement. κ values were interpreted as follows: <0.20, poor agreement; 0.21–0.40, fair agreement; 0.41–0.60, moderate agreement; 0.61–0.80, good agreement; and >0.80, excellent agreement.

Given the limited sample size and the descriptive focus of this study, no group comparisons, hypothesis testing, multivariable regression analyses, or survival analyses were performed. All statistical analyses were conducted using SPSS software (version 26.0). All statistical tests were two-sided, and a significance level of α = 0.05 was applied.

## 3 Results

### 3.1 Clinical characteristics of the patients

A total of 15 patients with pulmonary SMARCA4-deficient undifferentiated carcinoma were included in this study. The clinical characteristics of the patients are summarized in **Table 1**. The cohort predominantly consisted of middle-aged to elderly individuals, with a male predominance, and a subset of patients had a history of smoking. At initial presentation, most patients had compromised performance status, with ECOG scores predominantly 1–2.

**Table 1:** Clinical characteristics of individual patients with SMARCA4-deficient undifferentiated carcinoma of the lung.

Case	Age (years)	Sex	Smoking status	Presenting symptoms	ECOG	Clinical TNM stage	Distant metastasis	Metastatic sites	Treatment	Follow-up/outcome
1	67	Male	Never **smoker**	Cough	2	cT2N3M0, stage IIIB	No	–	Surgery + Chemotherapy	13 mo/Dead
2	68	Male	Current **smoker**	Bilateral leg edema	2	cT4N1M0, stage IIIA	No	–	Surgery + Chemotherapy	24 mo/Dead
3	52	Male	Current **smoker**	Organizing pneumonia	2	cT2bN0M0, stage IIA	No	–	Surgery + Chemotherapy	30 mo/Alive
4	75	Male	Current **smoker**	None	0	cT1cN0M0, stage IA3	No	–	Surgery	19 mo/Alive
5	67	Male	Current **smoker**	Cough, dyspnea	2	cT2N0M1c, stage IVB	Yes	Pleura, bone, liver	Best supportive care	7 mo/Dead
6	79	Male	Never **smoker**	Cough, sputum, back pain	3	cT2bN2M1b, stage IVA	Yes	Bone	Targeted therapy	3 mo/Dead
7	55	Male	Current **smoker**	Detected on physical exam	0	cT4NxM0, stage IIIA	No	–	Chemotherapy + Immunotherapy	16 mo/Dead
8	49	Female	Never **smoker**	Cough, hemoptysis	2	cT4N3M1c, stage IVB	Yes	Lymph nodes, bone	Chemotherapy	12 mo/Dead
9	68	Male	Former **smoker**	Detected on physical exam	1	cT1cN0M0, stage IA3	No	–	Microwave ablation	31 mo/Alive
10	74	Male	Never **smoker**	Cough	1	cT2aN0M0, stage IB	No	–	Immunotherapy	24 mo/Alive
11	65	Male	Current **smoker**	Cough, sputum	1	cT2N3M0, stage IIIB	No	–	Surgery + Chemotherapy	24 mo/Dead
12	70	Female	Never **smoker**	Cough	1	cT4N1M0, stage IIIA	No	–	Surgery + Chemotherapy	24 mo/Dead
13	77	Male	Current **smoker**	Cough, hemoptysis	2	cT2bN2M1c, stage IVB	Yes	Bone	Targeted therapy	3 mo/Dead
14	74	Female	Never **smoker**	Cough, hemoptysis	2	cT4NxM0, stage IIIA	No	–	Chemotherapy + Immunotherapy	6 mo/Dead
15	80	Male	Current **smoker**	Cough	2	cT2bN2M1c, stage IVB	Yes	Bone	Targeted therapy	3 mo/Dead

Clinical staging was based on imaging findings at initial presentation. In cases designated as Nx, nodal status was indeterminate on imaging, and stage grouping was assigned according to the available clinical data. Smoking status was categorized as current smoker (active smoker at diagnosis), former smoker (previous smoking history but not actively smoking at diagnosis), or never smoker (no documented history of tobacco use). Bold values indicate the smoking status categories defined in the table footnote: current smoker, former smoker, and never smoker.

According to the AJCC 8th edition lung cancer staging system, a substantial proportion of patients were diagnosed at locally advanced or advanced stages, with stage III and IV disease accounting for more than half of the cases. A subset of patients had evidence of distant metastases at initial diagnosis, most commonly involving the bone, adrenal glands, liver, or other organs.

Based on the predefined criteria described in the Methods section, synchronous metastases were identified in 5 patients at baseline staging. Among the 10 patients without baseline metastasis, distant metastases were subsequently documented during follow-up in all cases.

Pathologic confirmation was obtained in all patients by surgical resection or tissue biopsy, and loss of nuclear SMARCA4 (BRG1) expression was demonstrated by immunohistochemistry in every case.

### 3.2 CT imaging findings

All imaging evaluations were based on baseline chest CT scans obtained at initial diagnosis prior to any antitumor treatment, including non-contrast and contrast-enhanced CT in all 15 patients. CT imaging findings are summarized in **Table 2**.

**Table 2:** Imaging characteristics of SMARCA4-deficient undifferentiated carcinoma of the lung.

Imaging feature	Value
Tumor size, cm (median, range)	3.7 (0.6–9.5)
Central location	7/15 (46.7%)
Upper lobe involvement	9/15 (60.0%)
Spiculated margins	14/15 (93.3%)
Tumor necrosis	13/15 (86.7%)
Cavitation	7/15 (46.7%)
Marked or moderate enhancement	9/15 (60.0%)
Mediastinal invasion	10/15 (66.7%)
Vascular invasion	12/15 (80.0%)
Bronchial involvement	13/15 (86.7%)
Lymphadenopathy	13/15 (86.7%)
Conglomerated lymph nodes	11/15 (73.3%)
Pleural invasion	8/15 (53.3%)

Data are presented as number of patients (percentage) unless otherwise indicated. Imaging features were assessed on baseline CT images prior to any antitumor treatment.

On CT, tumors most commonly appeared as solid pulmonary masses, with a median maximum diameter of 3.7 cm (range, 0.6–9.5 cm). Lesions were distributed in both central and peripheral locations, with central tumors accounting for 46.7%. Most tumors had irregular margins, and spiculation was observed in 93.3% of cases. Marked intratumoral heterogeneity was a prominent feature, with tumor necrosis identified in 86.7% of patients, among whom 46.7% demonstrated cavitation.

On contrast-enhanced CT, the solid components of the tumors typically showed heterogeneous enhancement, with moderate-to-marked enhancement observed in 60.0% of cases. Invasive features involving adjacent structures were frequently observed, including mediastinal invasion (66.7%), vascular invasion (80.0%), and bronchial involvement (86.7%).

Lymph node metastases were common, with 86.7% of patients demonstrating hilar or mediastinal lymphadenopathy, among whom conglomerated lymph nodes were identified in 73.3%. In addition, 53.3% of patients showed pleural involvement or related imaging findings. A subset of patients demonstrated evidence of distant metastases on baseline CT or concurrent imaging studies.

Overall, these tumors frequently presented as irregularly marginated masses with marked internal necrosis, heterogeneous enhancement, and frequent involvement of adjacent structures and lymph nodes.

### 3.3 Interobserver agreement

Interobserver agreement between the two radiologists for major CT imaging features was consistently excellent. For key imaging findings, including tumor location (central vs. peripheral), intratumoral necrosis, mediastinal invasion, vascular invasion, and conglomerated lymph nodes, Cohen’s κ values ranged from 0.81 to 0.88, indicating a high level of reproducibility and reliability in CT image interpretation. Detailed results of the interobserver agreement analysis are provided in **Supplementary Table 1**.

### 3.4 Pathologic findings

All cases were confirmed using tissue specimens obtained by surgical resection or percutaneous biopsy, and diagnoses were established by routine histopathologic evaluation and immunohistochemical analysis. Histologically, the tumors were mainly composed of highly undifferentiated malignant cells with marked cellular pleomorphism and nuclear atypia. Variable degrees of tumor necrosis were observed, and definitive glandular or squamous differentiation was absent.

Immunohistochemical analysis demonstrated loss of nuclear SMARCA4 (BRG1) expression in all cases, while INI-1 expression was retained. Epithelial-related markers, including pancytokeratin (P-CK), TTF-1, Napsin A, p40, and CK5/6, were negative or only focally expressed. Neuroendocrine markers (synaptophysin, chromogranin A, and CD56) and NUT did not show specific expression in this cohort (see **Supplementary Table 2**).

These histomorphologic and immunohistochemical findings were consistent with the diagnosis of pulmonary SMARCA4-deficient undifferentiated carcinoma.

**Figure 1:**
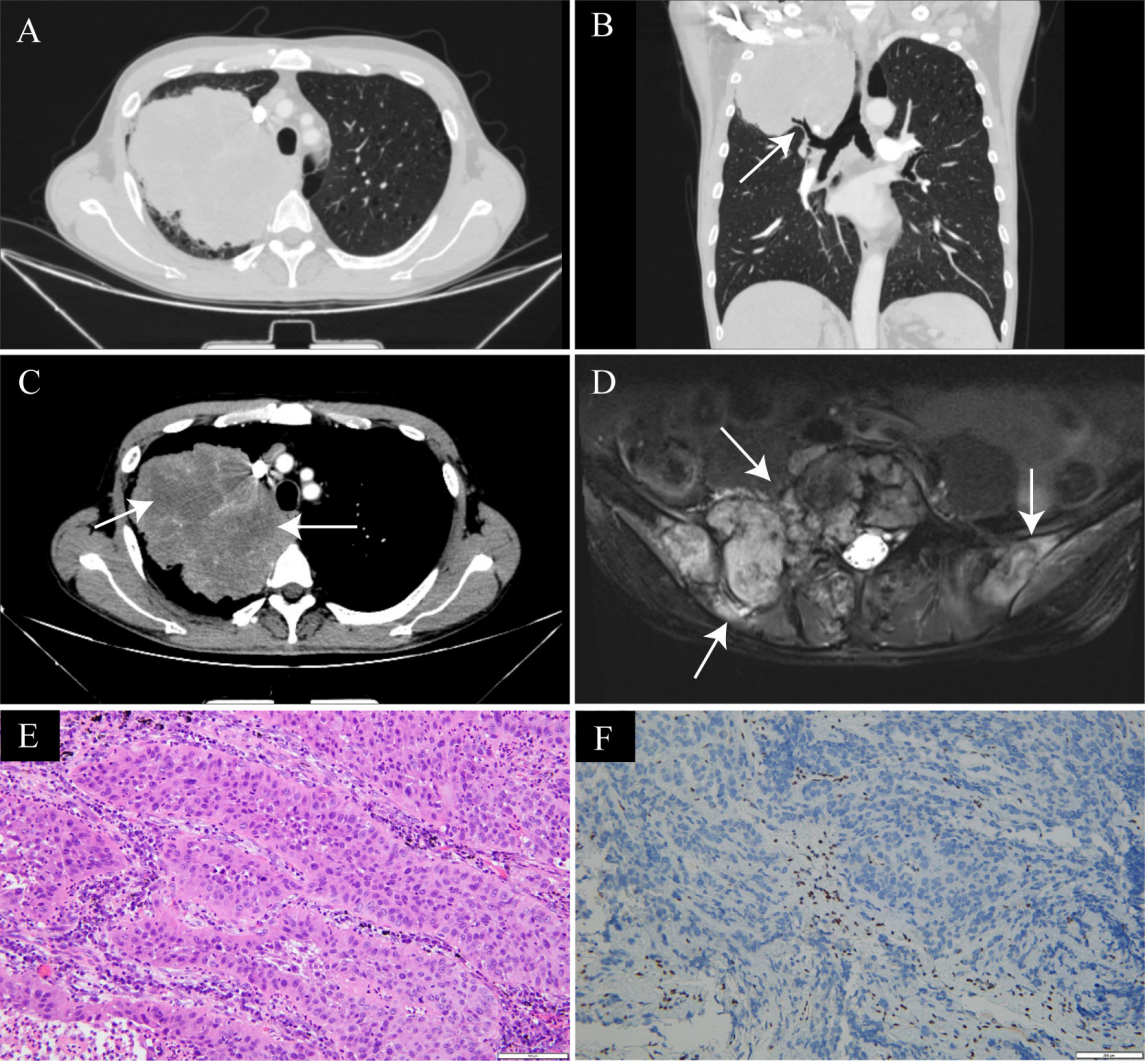
Representative imaging and pathological findings of pulmonary SMARCA4-deficient undifferentiated carcinoma in a 79-year-old man who presented with cough and hemoptysis for one month. **(A)** Axial chest CT lung window demonstrates a large solid mass in the right upper lobe with irregular margins. **(B)** Coronal reconstructed CT image shows invasion of the right upper lobe bronchus by the tumor (arrows). **(C)** Contrast-enhanced axial CT mediastinal window reveals markedly heterogeneous enhancement of the tumor, with hypoenhancing areas indicating intratumoral necrosis(arrows_)_. **(D)** Magnetic resonance imaging (MRI) demonstrates multiple destructive lesions involving the bilateral iliac bones and lumbar vertebrae (arrows), consistent with bone metastases. **(E)** Hematoxylin and eosin staining shows sheets of poorly differentiated tumor cells with marked nuclear atypia and focal necrosis (original magnification ×200). **(F)** Immunohistochemical staining reveals loss of nuclear SMARCA4 (BRG1) expression in tumor cells, confirming the diagnosis of SMARCA4-deficient undifferentiated carcinoma (original magnification ×200).

**Figure 2:**
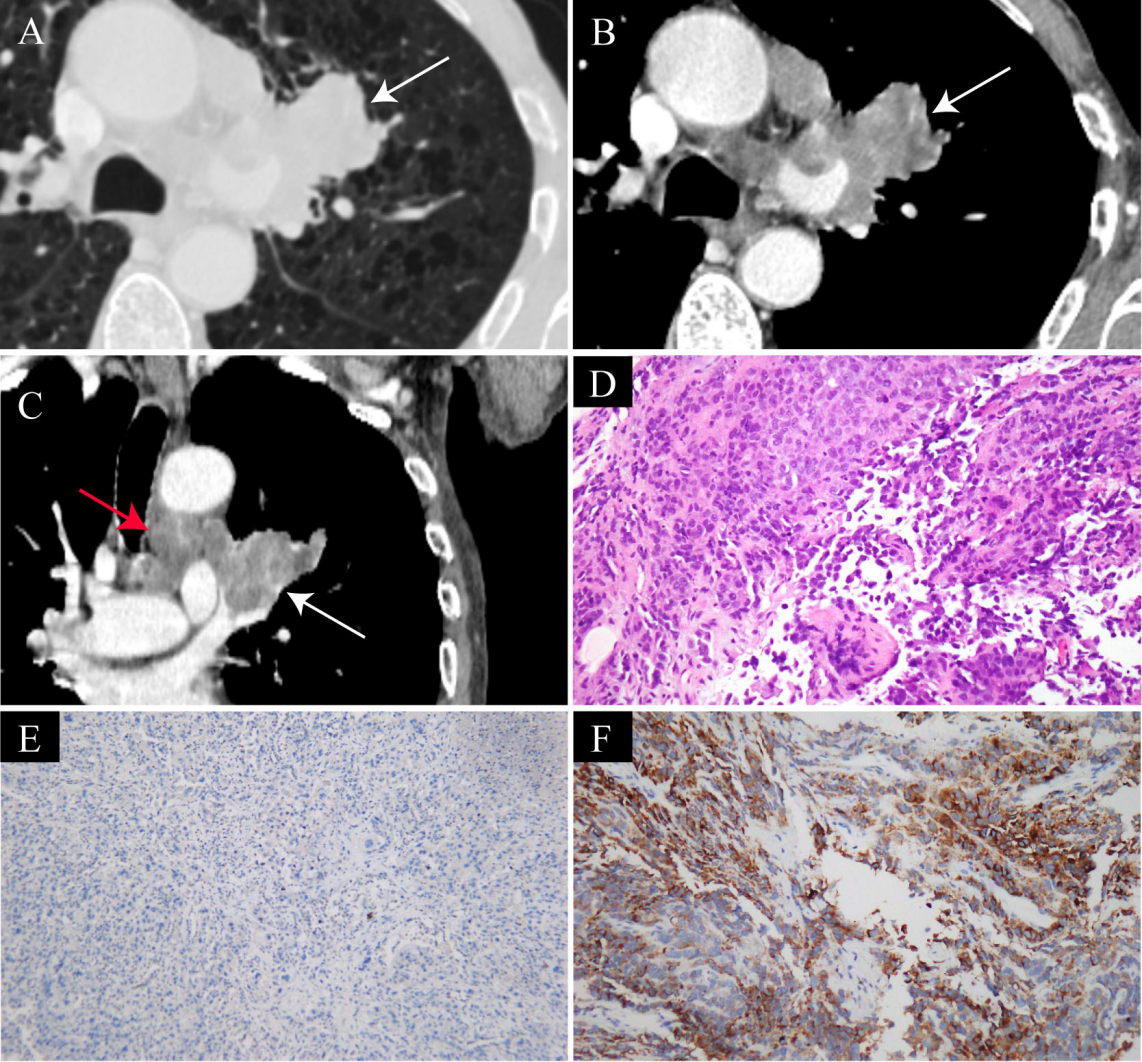
Aggressive imaging features and pathological confirmation of pulmonary SMARCA4-deficient undifferentiated carcinoma in a 55-year-old man who presented with hemoptysis for three months. **(A)** Axial chest CT lung window shows a left hilar mass (arrow), consistent with a centrally located tumor. **(B)** Contrast-enhanced axial CT mediastinal window demonstrates markedly heterogeneous enhancement of the tumor (arrow) with hypoenhancing areas indicating intratumoral necrosis, the mass is fused with enlarged hilar and mediastinal lymph nodes. **(C)** Coronal reconstructed CT image reveals invasion of the left upper lobe pulmonary artery by the tumor (white arrow), with multiple enlarged and conglomerated lymph nodes along the mediastinal great vessels (red arrow), suggesting extensive local invasion and lymph node metastasis. **(D)** Hematoxylin and eosin staining shows sheets of poorly differentiated tumor cells with marked nuclear atypia (original magnification ×200). **(E)** Immunohistochemical staining demonstrates loss of nuclear SMARCA4 (BRG1) expression in tumor cells (original magnification ×200), which represents the key diagnostic feature of pulmonary SMARCA4-deficient undifferentiated carcinoma. **(F)** Immunohistochemical staining shows absent or only focal expression of epithelial markers (original magnification ×200), supporting a dedifferentiated phenotype and aiding in the exclusion of other pulmonary malignancies.

Representative imaging and pathological findings are illustrated in **Figures 1**, **2**.

## 4 Discussion

### 4.1 CT imaging patterns of pulmonary SMARCA4-deficient undifferentiated carcinoma and comparison with previous studies

In the present study, we systematically analyzed baseline CT imaging findings in 15 patients with pulmonary SMARCA4-deficient undifferentiated carcinoma, with a particular focus on the co-occurrence of imaging features rather than isolated findings.

Previous studies on SMARCA4-deficient thoracic tumors have primarily consisted of case reports, small case series, and clinicopathologic analyses, in which imaging findings—such as necrosis, heterogeneous enhancement, and invasive growth—are typically described individually (2, 5, 7–10). However, these reports rarely address how these features coexist or whether they form a recognizable imaging pattern. In contrast, the current analysis provides proportion-based data on the combined occurrence of multiple aggressive CT features using standardized imaging definitions, thereby offering a more structured characterization of the imaging spectrum.

Marked intratumoral heterogeneity, particularly in the form of necrosis (86.7%) and cavitation (46.7%), was highly prevalent in this cohort. While previous literature has consistently noted tumor heterogeneity as a characteristic feature of SMARCA4-deficient tumors (5, 6, 11, 12), quantitative or proportion-based descriptions remain limited. By systematically evaluating these features, the present findings support the notion that necrosis and cavitation are not incidental observations but frequent components of the imaging presentation.

A similar pattern was observed for invasive features. Prior studies have described the aggressive nature of SMARCA4-deficient tumors, often highlighting local invasion or mediastinal involvement (2, 8, 13). However, the simultaneous involvement of multiple adjacent thoracic structures has not been consistently emphasized. In this cohort, bronchial, vascular, and mediastinal invasion frequently co-occurred, suggesting that multi-structure invasion may represent a characteristic imaging manifestation rather than an isolated finding. This observation provides a potential imaging correlate to the dedifferentiated and highly invasive biological behavior associated with SMARCA4 loss (1, 3, 6, 8, 14).

With respect to lymph node involvement, previous reports have documented nodal metastases but have provided limited detail regarding nodal morphology (2, 5, 11). In contrast, conglomerated lymphadenopathy was frequently observed in this study and often accompanied by extensive local invasion and intratumoral necrosis. This combined presentation may offer additional clues for radiologic suspicion, although it is not entirely specific.

Taken together, these findings suggest that pulmonary SMARCA4-deficient undifferentiated carcinoma is characterized not by a single dominant imaging feature, but by a reproducible combination of aggressive CT findings. This pattern-based perspective may provide practical value for radiologists by facilitating recognition of this rare entity in routine clinical settings.

### 4.2 Radiologic–pathologic correlation and implications for differential diagnosis

In this cohort, the CT manifestations were closely aligned with the markedly dedifferentiated pathological profile of pulmonary SMARCA4-deficient undifferentiated carcinoma. All tumors showed loss of nuclear SMARCA4 (BRG1) expression, lacked definitive glandular or squamous differentiation, and exhibited absent or only focal epithelial marker expression on immunohistochemistry (1, 4, 5, 11). Previous studies have established that this molecular and histopathologic profile is associated with aggressive tumor behavior (5, 7, 11, 15). The current findings further suggest that these biological characteristics are reflected on CT as marked heterogeneity and extensive invasive features.

With respect to radiologic–pathologic correlation, prior reports have described intratumoral necrosis as a common histologic feature in SMARCA4-deficient tumors (5, 7, 16, 17), but imaging-based characterization has largely remained qualitative. In the present analysis, low-attenuation regions and cavitary changes observed on CT were frequently identified and are likely to correspond to histologic necrosis. These findings support the concept that necrosis-related imaging features represent a reproducible component of the tumor’s radiologic presentation rather than incidental observations. Such correspondence may facilitate more consistent interpretation across radiologic and pathologic assessments.

A similar relationship was observed for invasive features. Previous pathological and molecular studies have emphasized the infiltrative growth pattern and high invasive potential of SMARCA4-deficient tumors (1, 16, 17). However, imaging correlates of this behavior have not been consistently characterized. In this cohort, imaging signs such as vascular encasement, bronchial narrowing or truncation, and obliteration of fat planes frequently co-occurred within the same lesion. This pattern of multi-structure involvement may represent a radiologic manifestation of the underlying infiltrative growth pattern and may help differentiate this entity from tumors that exhibit aggressive growth but more localized invasion.

From a differential diagnostic perspective, overlap with other high-grade thoracic malignancies remains a major challenge. Malignant lymphoma may also present as a large thoracic mass with associated lymphadenopathy; however, it more commonly demonstrates relatively homogeneous attenuation and less frequent direct invasion of adjacent airways or vessels on CT, as described in previous imaging studies (16, 18). Sarcomatoid carcinoma and poorly differentiated non–small cell lung cancer can also show aggressive local behavior; however, their imaging features are often more variable, and the simultaneous presence of marked intratumoral heterogeneity, extensive multi-structure invasion, and conglomerated lymphadenopathy is less consistently emphasized. Neuroendocrine tumors may exhibit rapid growth but often demonstrate different immunophenotypic profiles and, in many cases, a more homogeneous enhancement pattern (11, 18). In this context, consideration of combined imaging features rather than individual findings may improve radiologic discrimination.

Accordingly, when CT demonstrates a pulmonary mass with pronounced heterogeneity, extensive necrosis or cavitation, and simultaneous involvement of multiple adjacent thoracic structures, pulmonary SMARCA4-deficient undifferentiated carcinoma may be considered in the differential diagnosis within an appropriate clinical context. In such cases, targeted immunohistochemical testing, including assessment of SMARCA4 (BRG1) expression, may facilitate timely diagnostic confirmation and support multidisciplinary clinical decision-making (1, 5, 11, 17).

### 4.3 Clinical significance and therapeutic perspectives

The CT imaging features summarized in this study have practical implications for clinical assessment of pulmonary SMARCA4-deficient undifferentiated carcinoma. In this cohort, tumors frequently demonstrated pronounced local invasiveness and a high prevalence of lymph node and distant metastases at initial presentation, indicating that the disease is often at an advanced stage when first detected on imaging (1, 5, 11). These observations highlight the importance of CT not only in lesion detection but also in initial risk assessment and clinical decision-making.

From a clinical standpoint, when CT demonstrates a pulmonary mass with marked intratumoral heterogeneity, extensive necrosis or cavitation, and simultaneous involvement of the bronchi, vessels, and mediastinal structures, the likelihood of an aggressive and rapidly progressive malignancy should be considered (5, 8, 11, 16). In this cohort, such imaging features were frequently associated with advanced clinical stage and impaired performance status, potentially limiting eligibility for surgical intervention (1, 8, 11). Early recognition of this combination of imaging findings may therefore assist clinicians in anticipating treatment constraints and in prioritizing systemic or non-surgical therapeutic strategies when appropriate.

Recent studies suggest that SMARCA4-deficient tumors may show limited responsiveness to conventional chemotherapy, whereas a subset of patients may benefit from immunotherapy-based approaches (2, 19, 20). Although treatment outcomes were not formally analyzed in the present study, the imaging characteristics observed—particularly tumor burden, degree of necrosis, and extent of invasion—may reflect underlying tumor biology. In this context, CT findings may provide supplementary information when clinicians evaluate treatment options, although prospective validation is required.

In addition, radiologists play a key role in raising early diagnostic suspicion. When imaging findings suggest a highly aggressive undifferentiated pulmonary malignancy, explicit recommendation for further pathological evaluation, including SMARCA4 (BRG1) immunohistochemical testing, may facilitate timely diagnosis and reduce delays in appropriate management (2). From a practical perspective, clear description of key imaging features in radiologic reports may improve communication within the multidisciplinary team and support more efficient diagnostic pathways.

Overall, careful evaluation of combined CT imaging features may assist in early clinical suspicion, guide diagnostic work-up, and inform therapeutic planning for this rare but highly aggressive tumor.

### 4.4 Study limitations and future directions

Several limitations of this study should be acknowledged. First, this was a single-center retrospective study with a relatively small sample size, which may introduce selection bias and limit the generalizability of the findings. Given the rarity of pulmonary SMARCA4-deficient undifferentiated carcinoma, the present results should be interpreted with caution and considered as preliminary observations. Validation in larger, multicenter cohorts will be necessary to confirm the reproducibility and broader applicability of the imaging findings (5, 8, 21, 22).

Second, this study was primarily based on baseline CT imaging, and longitudinal imaging changes during or after treatment were not systematically analyzed. In addition, potential associations between imaging features and clinical outcomes, including prognosis and treatment response, were not evaluated. Future studies incorporating follow-up imaging and outcome data may help clarify the clinical relevance of these imaging features (23, 24).

Third, although interobserver agreement for key imaging features was good to excellent, qualitative image interpretation inevitably involves a degree of subjectivity. Furthermore, quantitative imaging parameters, such as attenuation measurements, were not systematically assessed due to variations in imaging protocols inherent to the retrospective design. Future investigations may benefit from more standardized imaging acquisition and analysis, as well as from the incorporation of quantitative approaches, including radiomics and artificial intelligence–based methods, to provide more objective characterization of tumor heterogeneity and invasiveness (25, 26).

Finally, the lack of a control or comparison group limits the ability to determine the specificity of the observed imaging features relative to other high-grade thoracic malignancies. Although indirect comparisons with previous studies were performed, prospective studies with appropriate comparator cohorts are needed to further evaluate the diagnostic value of combined imaging features.

Future research should focus on multicenter validation of imaging findings, integration of longitudinal imaging with clinical outcomes, and exploration of imaging–pathologic–molecular correlations. Such efforts may help refine the role of CT in the diagnostic assessment and clinical management of this rare and aggressive tumor.

## 5 Conclusions

In this cohort of 15 patients, pulmonary SMARCA4-deficient undifferentiated carcinoma most commonly presented on baseline CT as a heterogeneous pulmonary mass with spiculated margins (14/15, 93.3%), intratumoral necrosis (13/15, 86.7%), cavitation (7/15, 46.7%), bronchial involvement (13/15, 86.7%), vascular invasion (12/15, 80.0%), mediastinal invasion (10/15, 66.7%), and conglomerated lymphadenopathy (11/15, 73.3%). Rather than any single imaging sign, the combined presence of marked heterogeneity, necrosis or cavitation, multi-structure invasion, and bulky nodal disease supports a reproducible aggressive CT imaging profile at presentation. Although these findings are not individually specific, their co-occurrence may help raise suspicion for pulmonary SMARCA4-deficient undifferentiated carcinoma over other aggressive thoracic malignancies with overlapping imaging features, such as sarcomatoid carcinoma, poorly differentiated non–small cell lung cancer, and malignant lymphoma, thereby prompting earlier consideration of targeted immunohistochemical confirmation. Future multicenter studies with larger cohorts are needed to validate this CT imaging profile, clarify its differential diagnostic value, and further explore its associations with molecular features, treatment response, and clinical outcomes.

## Data Availability

The raw data supporting the conclusions of this article will be made available by the authors, without undue reservation.
