# Supplementary material for: CT imaging features of pulmonary SMARCA4-deficient undifferentiated carcinoma: a retrospective case series
Source: Front Oncol. 2026 May 18;16:1822031. doi: 10.3389/fonc.2026.1822031 (PMC13222827; doi:10.3389/fonc.2026.1822031)
Supplement: Supplementary file 1 [file Table1.docx]

Supplementary Table S1. Interobserver agreement analysis of imaging features

Two radiologists independently evaluated major imaging features on baseline CT images. Interobserver agreement was assessed using Cohen’s κ statistics.

| Imaging feature | κ value | 95% CI | Agreement category |
| --- | --- | --- | --- |
| Tumor location  (central vs peripheral) | 0.82 | 0.65–0.94 | Excellent |
| Tumor necrosis  (present vs absent) | 0.85 | 0.70–0.96 | Excellent |
| Mediastinal invasion | 0.81 | 0.63–0.93 | Excellent |
| Vascular invasion | 0.88 | 0.74–0.97 | Excellent |
| Confluent lymph nodes | 0.84 | 0.68–0.95 | Excellent |

κ values were interpreted as follows: <0.20 poor, 0.21–0.40 fair, 0.41–0.60 moderate, 0.61–0.80 good, and >0.80 excellent agreement.

Supplementary Table S2. Immunohistochemical findings of SMARCA4-deficient undifferentiated carcinoma of the lung

| Case | SMARCA4 (BRG1) | P-CK | Vimentin | CK7 | CK20 | Villin | TTF-1 | Napsin A | CK5/6 | p40 | p63 | CD56 | CgA | Syn | INSM1 | INI-1 | NUT | Ki-67 (%) |
| --- | --- | --- | --- | --- | --- | --- | --- | --- | --- | --- | --- | --- | --- | --- | --- | --- | --- | --- |
| 1 | Loss | + | + | +  (focal) | - | - | + | - | - | - | - | - | - | - | - | Retained | - | ~60 |
| 2 | Loss | + | - | + | - | - | - | - | - | - | - | - | - | - | - | Retained | - | ~75 |
| 3 | Loss | + | - | - | - | - | - | - | - | - | - | - | - | - | - | Retained | - | ~5 |
| 4 | Loss | + | - | - | - | - | - | - | + | + | - | - | - | - | - | Retained | - | ~30 |
| 5 | Loss | + | - | + | - | - | - | - | + | + | - | - | - | - | - | Retained | - | ~20 |
| 6 | Loss | - | + | - | - | - | - | - | - | - | - | - | + | - | - | Retained | - | ~65 |
| 7 | Loss | + | - | + | - | - | - | - | - | - | - | - | - | - | - | Retained | - | ~80 |
| 8 | Loss | + | + | +  (focal) | - | - | + | - | - | - | - | - | - | - | - | Retained | - | ~90 |
| 9 | Loss | + | - | + | - | - | + | - | + | + | + | - | - | - | - | Retained | - | ~70 |
| 10 | Loss | - | - | - | - | - | - | - | - | - | - | - | - | - | - | Retained | - | ~70 |
| 11 | Loss | + | - | - | - | - | - | - | - | - | - | - | - | - | - | Retained | - | ~40 |
| 12 | Loss | + | - | - | - | - | - | - | - | - | - | - | - | - | - | Retained | - | ~50 |
| 13 | Loss | + | - | - | - | - | - | - | - | - | - | - | - | - | - | Retained | - | ~80 |
| 14 | Loss | + | - | - | - | - | - | - | - | - | - | - | - | - | - | Retained | - | ~30 |
| 15 | Loss | + | - | - | - | - | - | - | - | - | - | - | - | - | - | Retained | - | ~90 |

Occasional focal positivity for individual epithelial or neuroendocrine markers was observed in some cases; however, no case showed diffuse expression of lineage-specific markers. Vimentin expression was present in a subset of tumors, consistent with the dedifferentiated phenotype. The Ki-67 proliferation index showed marked intertumoral variability, reflecting biological heterogeneity.
